# Awake Mouse fMRI and Pupillary Recordings in the Ultra-High Magnetic Field

**DOI:** 10.3389/fnins.2022.886709

**Published:** 2022-07-06

**Authors:** Hang Zeng, Yuanyuan Jiang, Sandra Beer-Hammer, Xin Yu

**Affiliations:** ^1^High-Field Magnetic Resonance Department, Max Planck Institute for Biological Cybernetics, Tuebingen, Germany; ^2^Graduate Training Centre of Neuroscience, International Max Planck Research School, University of Tuebingen, Tuebingen, Germany; ^3^Athinoula A. Martinos Center for Biomedical Imaging, Harvard Medical School, Massachusetts General Hospital, Charlestown, MA, United States; ^4^Department of Pharmacology, Experimental Therapy, and Toxicology, Interfaculty Center of Pharmacogenomics and Drug Research (ICePhA), University of Tüebingen, Tübingen, Germany

**Keywords:** awake mouse fMRI, visual stimulation, longitudinal training, headpost implantation, pupillometry

## Abstract

Awake rodent fMRI is becoming a promising non-invasive brain imaging module when investigating large-scale brain function given behavioral tasks. Previous studies have either applied sedatives during scanning or pre-treatment of anesthetics, e.g., isoflurane, to reduce the motion of animals, which could confound the brain function of “awake” states in rodents. Here, we have established a long training awake mouse fMRI-pupillometry paradigm/setup without the initial use of anesthesia. To validate the awake mouse fMRI platform, evoked BOLD-fMRI was performed to identify brain activation in the visual cortex, dorsal lateral geniculate nuclei, and superior colliculus. Furthermore, pupil signal fluctuation was investigated during scanning, showing a less dilated pupil after 5–8 weeks of intermittent training. Thus, using the awake mouse fMRI with real-time pupillometry provides a longitudinal functional mapping tool to study fully conscious mice.

## Introduction

Since the late 1990's, animal models have been subjected to awake functional magnetic resonance imaging (fMRI) (Lahti, 1998). In the past two decades, numerous biological techniques (e.g., optogenetics, transgenic, pharmacological manipulations, etc.) have been combined with awake animal fMRI for function–behavior studies (Desai et al., 2011; Ferris et al., 2014; Behroozi et al., 2020). However, it remains challenging to interpret the fMRI signal acquired in awake brain states due to unmonitored stress levels and motion-related image artifacts during scanning. To minimize these confounding effects, various habituation protocols and restrained-motion schemes have been introduced. In particular, to achieve better MRI shimming and secure the animals into MRI apparatus, short-period pre-scanning anesthesia or sedative treatment were applied in many imaging modalities. We summarized the small animal awake fMRI studies since the year 2000 in Supplementary Table 1. Despite the clearance of anesthetics from the circulatory systems of animals after a waiting period, the global long-lasting effects on neurovascular coupling are not fully addressed (Makaryus et al., 2011; Thrane et al., 2012; Tsurugizawa et al., 2020). To establish an anesthetic-free awake animal fMRI platform remains to be further optimized.

With the ever-growing exploration of awake animal fMRI, customized restraining apparatus as well as longitudinal training procedures, have been designed to circumvent confounding effects, such as animal stress and motion artifacts. Conventional training schemes have varied periods from 10 min in the early phase to 60–90 min in the later phase for consecutive 7–10 days (Supplementary Table 1). Besides heart/respiration rate monitoring, previous efforts have been applied to measure plasma corticosterone to assess the stress level during training, but the real-time scanning-induced depression or a state of helplessness has seldom been fully investigated (King et al., 2005; Febo, 2011; Low et al., 2016). It became clear that minimizing and attenuating the stress effect of awake rodents during scanning is a critical step for function–behavior studies.

Previous awake rodent fMRI studies present the unique functional mapping capability of genetically engineered mouse models. For instance, Tsurugizawa et al. (2020) have performed fMRI to map the mouse brain in a disease model of autism, identifying white matter anomalies and evaluating pharmacological treatments based on the behavioral outcomes. Takata et al. (2018) have applied optogenetic fMRI to different transgenic mouse lines to study the astrocyte dynamic features. Interestingly, pupillometry, a non-invasive indicator of altered arousal (Bradley et al., 2008), vigilance (Ebitz et al., 2014), emotion (Partala and Surakka, 2003), and cortical states (Reimer et al., 2014), has been combined with fMRI studies of human and animal brains (Murphy et al., 2014; Schneider et al., 2016, 2018; Pais-Roldan et al., 2020; Sobczak et al., 2021a). Importantly, there are quite a few literatures to show the stress linkage with pupil size in fish (Slavik et al., 2020) and humans (Schneider et al., 2016, 2018; Schneider, 2018), as well as the cognitive (attention) demand with pupil size changes. The measurement of concurrent fMRI and pupil dynamics in awake mice remains to be established to further decipher the global functional basis underlying different pupil dynamic spectrums(Sobczak et al., 2021a,b).

In the present study, we aim to establish an fMRI-pupillometry platform to study the brain function of awake mice. An 8-week intermittent training protocol was designed to habituate head-fixed mice on the bench and in the magnetic bore, allowing longitudinal brain functional mapping. To validate the awake mouse fMRI platform, we also performed visual stimulation to map evoked BOLD signals along with the visual system, demonstrating the feasibility of awake mouse fMRI with pupillometry during scanning.

## Materials and Methods

### Animals

All the experimental procedures were approved by the state authority (Regierungspräsidium, Tübingen, Baden-Württemberg, Germany) and conducted in accordance with the guidelines. Nine male C57BL/6 mice (Charles River Laboratory) were employed in this project and two were excluded due to the headpost cracking during training. Animals were habituated individually at a 12 h−12 h light–dark cycle (light on from 8 a.m. to 8 p.m.) with food and water *ad libitum*, starting at age 10–15 weeks.

### Behavioral Handling and Training

A 3D printed headpost and customized training boxes (Schwarz et al., 2010) have been designed for acclimating the animals (Supplementary Figure 1). Briefly, intermittent habitual procedures were applied to train animals for 5–8 weeks before fMRI experiments. The training procedure includes: (1) mice were quietly settled in a comfortable, natural posture in the training box; (2) mice were immobilized with a headpost in the training box; (3) mice were exposed to the loud scanning noise in the scanning bore of a 14.1T scanner; (4) the slow fluctuation of pupil dynamics was recorded; and (5) training was continued until less/no urination or defecation (fecal boli <10) during imaging/training. Only mice that have good performance (mostly quiet, little urination or defecation, slow pupil dynamics) will continue with the next phase of imaging.

Mice were first handled in the experimenter's gloved hands for approximately 5 min/section until they exhibited grooming behaviors. Then, they were fixed through a headpost to the training box. The training period was increased from 10–15 min/day to 60 min periodically (1–2 days of rest after each training). The increment protocol in the training time is as follows: 10–15 min/session at the very beginning (1 week), and then 20–30 min for the next training session (2–3 weeks), 30–40 min (3–4 weeks), 40–50 min (4–5 weeks), 50–60 min (5–8 weeks). Mice body posture and movements were monitored as an indicator of training efficacy. A video camera with infra light was used to continuously monitor pupil dynamics. One week after surgery, mice were first acclimated in the training box without head fixation (~2 weeks) in the scanner, and then mice were head-fixed through headpost and kept inside the MR scanner while the scanner was running with designated sequences. Between each training section, mice rested for 1–2 days, during which the training process was maintained for at least 5 weeks prior to the actual MRI experiment (Supplementary Video 1). Animals received sucrose water several times between each training section (Supplementary Video 2). After finishing the last training section, the animals were imaged in the 14.1T MRI scanner for 24 h.

### Headpost Implantation

Mice were first anesthetized by isoflurane (2% for induction; 1–1.3% for surgery in a mixture of 30% oxygen in the air). The depth of anesthesia was checked by the lack of pedal withdrawal to a firmly pinched hind toe or foot, or the lack of head movement to a firmly pinched ear. The temperature was maintained at 37°C using a controlled heating pad. A small amount of ophthalmic ointment was placed in each eye to protect it from drying. Then, mice were secured to a stereotaxic frame by two earpieces and a bite bar. The skull was shaved and disinfected. After exposing the skull with a surgical knife, the soft tissue and periosteum were carefully removed and cleared with Kerr Gel Etchant (37.5% phosphoric acid, OptiBondTM FL) for 15 s. Then, the skull surface for implantation was prepared following the procedure as suggested by the manufacturer. We applied dental cement (Charisma flow) to fix the headpost above the skull. The remaining incision area was sutured and disinfected with antiseptics. Painkillers and antibiotics were administered to relieve pain and inflammation immediately after finishing the surgery. Animals were maintained on a heated blanket until fully recovered from the anesthetic effect (15–30 min) and were placed back in their original cages. Post-surgical observations were documented for a minimum of five consecutive days following the surgery (twice a day for 5 days and then daily). Wound recovery was monitored daily in the first week after surgery to ensure that there was no infection and no sutures off (Supplementary Video 3).

### Visual Stimulation Paradigms and Pupil Diameter Recording

To investigate BOLD responses under visual stimulation in awake mice. A customized LED light Matrix System (P160104 LED matrix, 470 nm) for visual stimulation (Supplementary Video 4) was triggered by the echo-planar imaging (EPI) sequence and was controlled with the master 9 system (Master-9, A.M.P.I). A block design paradigm of 4s-on-16s-off in 10 epochs for each imaging session was conducted. The light brightness (~6 μW) and flickering frequency (~5 Hz) were also controlled within the system. To monitor the chest movement, a pressure sensor (Graseby Sensor, Medicare Health & Living Ltd) was attached under the animals' abdomen, from which the motion effect was detected by the BIOPAC acquisition system (Biopac Systems Inc., USA).

Customized and copper-sealed MRI-compatible cameras (RS-OV7949-1818, Conrad Electronic SE) were applied for monitoring head movements and acquiring pupillometry videos during imaging (32 bites/pixel, 60 frames/s, and resolution: 1920 × 1080). The camera was held on a home-built adjustable holder (Supplementary Figure 2). A movable infrared LED light (850 nm) was positioned beside the eyes of mice for pupil recording.

### Magnetic Resonance Imaging

MRI images were acquired using a Bruker Avance III System (Bruker BioSpin, Ettlingen, Germany) with a 14.1-T superconducting magnet with a 12 cm diameter gradient providing 100 G/cm with a 150 μs rise time. A custom-made transceiver surface coil (8 mm in diameter) covering the whole brain of mice was applied for imaging. Functional scans were acquired using a 2D echo-planar imaging (EPI) sequence, with the following parameters: TR = 1 s, TE = 8 ms, FOV = 12 × 12 mm, 40 × 40 acquisition matrix, 24 slices, 0.3 × 0.3 × 0.5 mm^3^ resolution, 210 TRs total time. The anatomical RARE (rapid acquisition with relaxation enhancement sequence) images were acquired using the same geometry as functional scans with the following parameters: TR = 2.5 s, TE = 7 ms, FOV = 12 × 12 mm, 96 × 96 acquisition matrix, 24 slices, 0.125 mm^2^ in-plane resolution, 0.5 mm slice thickness, 6 × RARE factor, 4 repetitions. The magnetic field homogeneity was corrected using the FASTMAP scout. A well-trained mouse was used to adjust the magnetic field homogeneity, which was also used as a reference for imaging other mice. The duration for multi-order shimming and positioning adjustment is ~17 min.

### Data Analysis

All fMRI data were analyzed using the AFNI software package [Analysis of Functional NeruoImage, https://afni.nimh.nih.gov/(R. W. Cox, 1996)). The preprocessing steps include (1) motion correction (3dvolreg), (2) co-registration to the anatomical images (align_epi_anat.py), (3) segmentation (3dSkullStrip, using a brain mask), (4) spatial smoothing (3dmerge, using a Gaussian kernel of FWHM = 0.2mm), (5) image normalization, and (6) voxel-wise linear regression analysis to calculate BOLD statistical maps (3dDeconvolve). This final step applied the hemodynamic response function (HRF) model: BLOCK (d, p), which is convolved with a square wave of duration “d” and made to have a peak amplitude of “*p*” (the amplitude of the basis function and usually set to 1).

The activated ROIs in functional maps were extracted and detrended for baseline shift. The baseline signal of EPI images was normalized to 100 for later multiple trial statistical analysis. To characterize the signal changes, custom-written MATLAB (MathWorks, Natick, USA) scripts were used to average the BOLD signal time series from each epoch and convert them to percentage change based on the signal intensity of 6 TRs prior to stimulation. The anatomical MRI images were registered to a template across animals for later image processing by applying the 3dAllineate function.

For pupil data preprocessing, all the pupillometry videos were cropped and processed using the DeepLabCut toolbox (Mathis et al., 2018). A mouse pupil network was built in this project to extract the pupil size from each video (Supplementary Video 4). The *network* was generated by using 5,670 frames from 27 videos (5 mice). Through this specially built network, four boundary dots with traced coordinates (*x*_i_,y_i_), *i* = 1, …, 4, were used to calculate the pupil diameter (22):


(1) $$\begin{array}{l} diameter\;=\;\frac{1}{2}\times (\sqrt{{\left(x2-x1\right)}^{2}+{\left(y2-y1\right)}^{2}}\;\\ \;+\sqrt{{\left(x4-x3\right)}^{2}+{\left(y4-y3\right)}^{2}}) \end{array}$$

The resting-state pupil data were collected and applied for statistical analysis to monitor the physiological states. One-way ANOVA was applied to estimate the animals' pupil data in 1^st^, 5th, and 8^th^ weeks of training.

## Results

### Establishment of the fMRI-Pupillometry Training Paradigm and Platform

**Figure 1:**
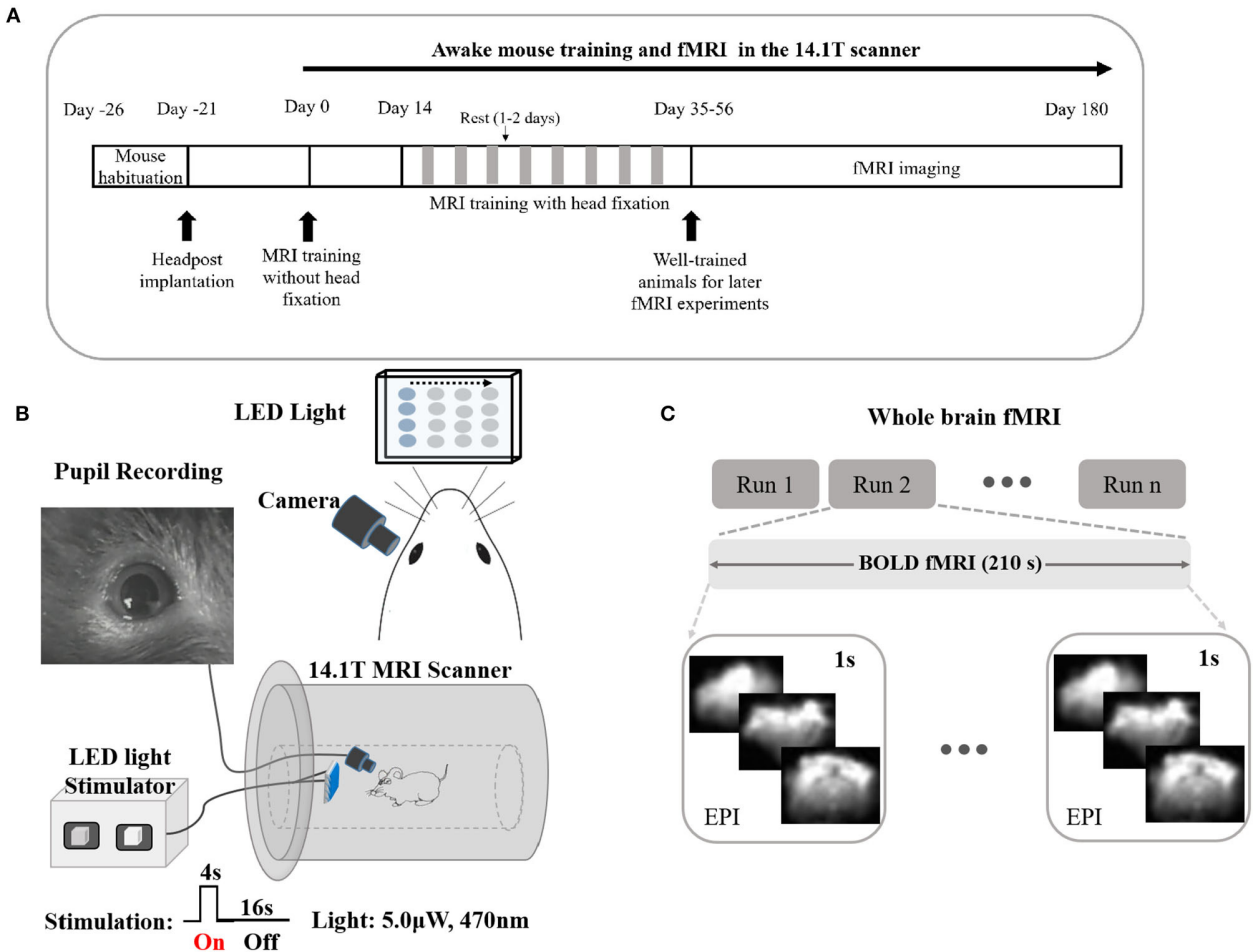
Long training awake fMRI-pupillometry paradigm/setups in rodents without the initial use of anesthesia. **(A)** The intermittent restraint and training protocol. **(B)** Schematics of visual stimulation paradigms and pupil recordings with fMRI. **(C)** Designs of whole-brain fMRI modalities.

The schematics of the fMRI-pupillometry platform and awake mouse training scheme are shown in Figure 1A. Different from the pervious “mock scanner”-based restraining system, the animals were exposed to the real scanner noise fixed in a home-designed restraining system (Supplementary Figure 1). There were no anesthetics applied during the entire training and imaging sections. After 5–8 weeks of training, mice were adapted to the restraining system and showed an inclination to keep an “imaging posture” even without head fixation compared with the animals trained in the short time period (Supplementary Videos 5–8). The chest movements of mice with longer training periods showed less motion, indicating a quantitative measurement of well-trained mice during fMRI (Supplementary Figure 3).

An infrared camera with copper shielding and a blue LED light matrix was implemented to record the pupil diameter and to deliver visual stimulation, respectively (Figure 1B, Supplementary Video 4, and Supplementary Figure 2). The flickering light stimulus (0–6 μW, 0–10 Hz) was delivered during imaging sessions. Whole-brain fMRI datasets were acquired in both evoked and resting states (Figure 1C).

### Head Motion Assessment

**Figure 2:**
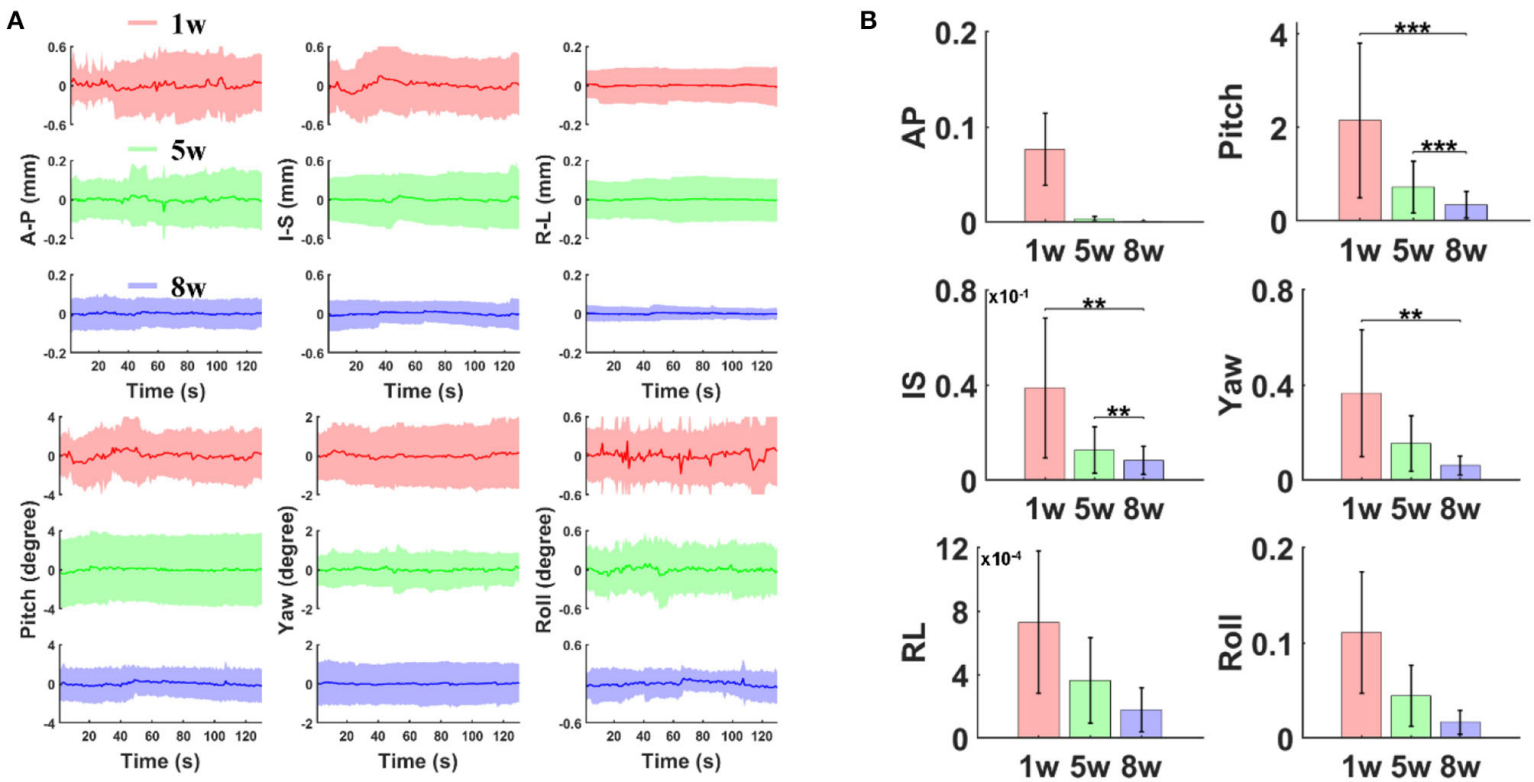
The estimated motion parameters during intermittent acclimation from different weeks. **(A)** Averaged time course of translation displacement (A–P: anterior–posterior; I–S: inferior–superior; R–L: right–left) and rotations (pitch, yaw, and roll) after different weeks of training (first, fifth, eighth week; shaded regions: mean ± S.D, *n* = 6 animals). **(B)** Variance of translation and rotation time course (** *p* < 0.01, *** *p* < 0.001 by Bonferroni *post-hoc* test, error bars, S.E.M., *n* = 6 animals).

Head motion assessment was calculated and compared after different periods of training sessions (1st, 5th, and 8th weeks). Based on the registration process of 3D fMRI datasets acquired at different training phases, six motion parameters (three translation displacements: anterior–posterior (AP), right–left (RL), inferior–superior (IS), and three rotational displacements of roll, pitch, and yaw) were processed (Figure 2). The rotation and movement time courses were gathered from six mice. The average time courses of translation and rotation displacements corresponding to the 1st, 5th and 8th weeks are shown in Figure 2A (A–P, 0.04 ± 0.03 vs. 0.01 ± 0.01 vs. 0.003 ± 0.002 mm; I–S, 0.04 ± 0.03 vs. 0.01 ± 0.01 vs. 0.01 ± 0.009 mm; R–L, 0.003 ± 0.002 vs. 0.002 ± 0.002 vs. 0.002 ± 0.001 mm; Pitch, 0.27 ± 0.22 vs. 0.11 ± 0.09 vs. 0.14 ± 0.08 degree; Yaw, 0.08 ± 0.05 vs. 0.05 ± 0.04 vs. 0.02 ± 0.01 degree; Roll, 0.05 ± 0.05 vs. 0.03 ± 0.02 vs. 0.03 ± 0.02 degree; mean ± SD). The variance of those motion parameters from each individual trial were extracted and compared (Figure 2B, one-way ANOVA, A–P, *F* (2, 47) = 3.26, *P* = 0.047; I–S, *F* (2, 47) = 6.94, *P* = 0.002; R–L, *F* (2, 47) = 2.36, *P* = 0.106; Pitch, *F* (2, 47) = 8.63, *P* = 0.0006; Yaw, *F* (2, 47) = 5.64, *P* = 0.006; Roll, *F*(2, 47) = 2.6, *P* = 0.085).The estimated framewise displacement during intermittent acclimation from different weeks after different weeks of training is shown in Supplementary Figure 4. Head motion was reduced along all planes following the training protocol.

### Real-Time Pupil Dynamics in Resting-State Awake Mice fMRI

**Figure 3:**
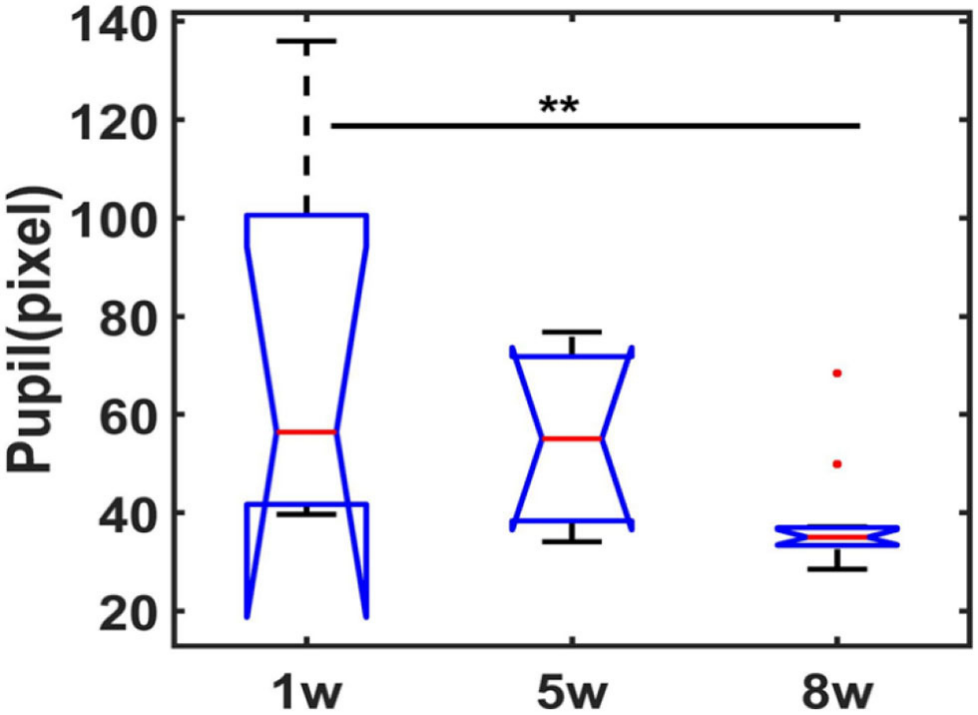
Statistical analysis of pupil dynamics in resting-state following different weeks of training. Boxplot graph represents the pupil diameter changes (central red line is the median, 1w: 56.42, 5w: 55.08s, 8w: 35.04; interquartile range 25th and 75th percentiles denote the maximum and minimum, outliers are shown by red dots, ** *p* < 0.01, by Bonferroni *post-hoc* test, mean trials = 9 each week session, *n* = 3 animals).

Under isoluminance, the pupil dynamic changes were recorded at different phases of the training protocol when exposed to scanner noise during scanning. Figure 3 shows the statistical comparisons of raw pupil data in the 1st, 5th, and 8th weeks of training (3 mice, one-way ANOVA, *F* (2, 27) = 5.92, 1st vs. 8th = 0.0078, Bonferroni *post-hoc* test). These results indicate that the animal pupil dynamics dilated at the very beginning of training and subsequently reduced at later training time periods, indicating a more relaxed state of mice during fMRI. Meanwhile, the resting state pupil dynamics were plotted as a function of time, as well as the light exposure-induced pupil constriction (Supplementary Video 4), indicating the feasibility of awake mouse fMRI with real-time pupillometry.

### Visual Stimulation-Induced BOLD Responses in Awake Mice

**Figure 4:**
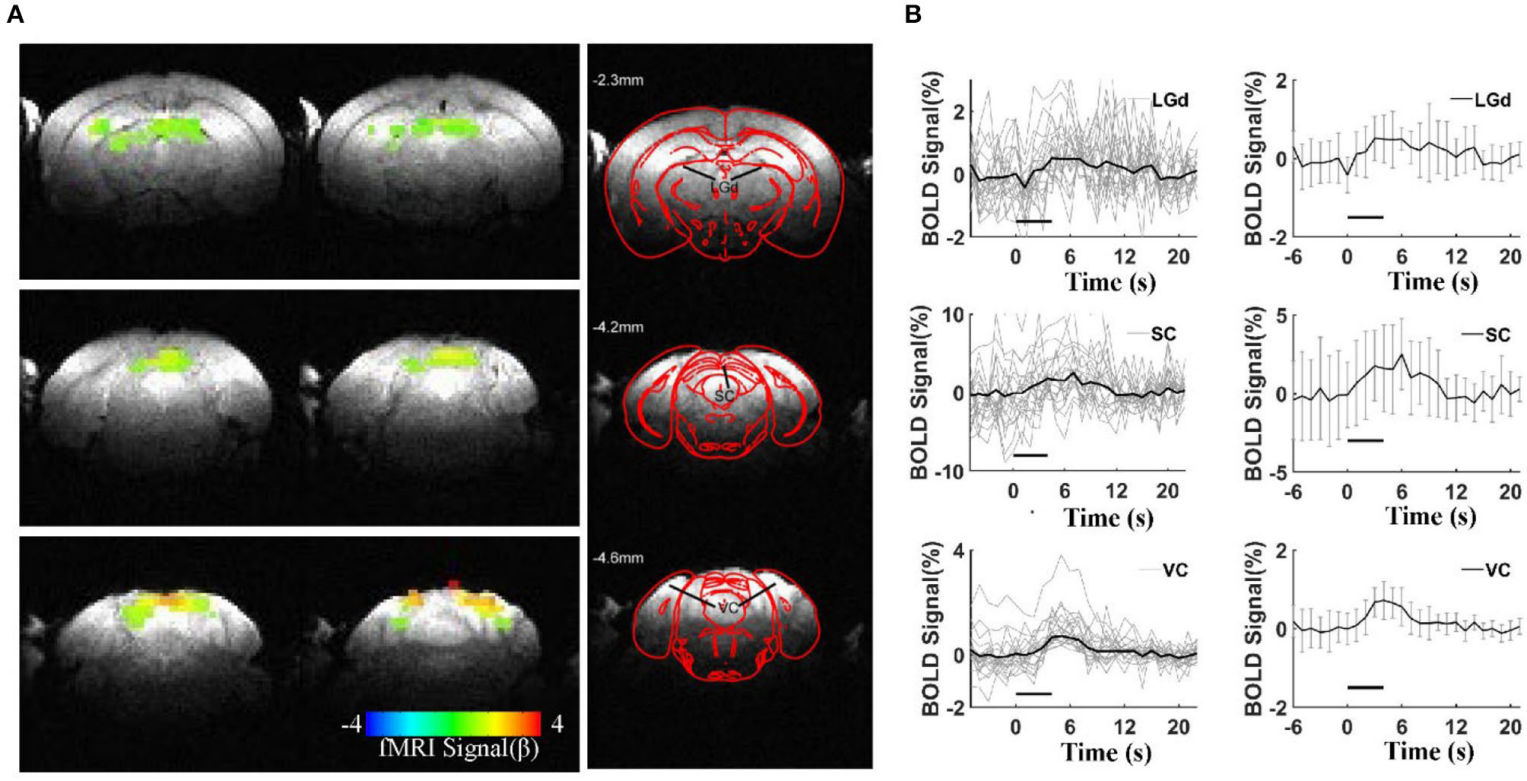
BOLD responses under visual stimulation. **(A)** Representative color-coded BOLD-fMRI maps overlaid on anatomical images, showing dorsal lateral geniculate nucleus (LGd), superior colliculus (SC), and visual cortex (VC) activations in visual circuit-related regionswith a statistical threshold (*p* < 0.001, cluster size >20 voxels). **(B)** Averaged BOLD signals percentage changes upon visual stimulations in the visual circuit-related regions, left column: the individual epoch of signal changes from all the animals, right column: the mean BOLD signal changes (6 mice, error bars, mean ± SD, horizontal bars, duration of light stimulation).

To validate the awake mouse fMRI-pupillometry platform, BOLD fMRI signals were acquired under visual simulation. Three main brain regions along the visual pathway showed positive BOLD activation (*p* < 0.001): the visual cortex (VC), superior colliculus (SC), and dorsal lateral geniculate nucleus (LGd) (Figure 4A). Averaged time courses of BOLD signal changes of the three regions from different animals (VC, SC, and LGd) showed a maximum of 0.72, 2.5, and 0.5%, respectively (Figure 4B). The representative raw RARE and EPI images are shown in Supplementary Figure 5. Similar to previous fMRI studies(Niranjan et al., 2016; Desjardins et al., 2019; Tsurugizawa et al., 2020; Dinh et al., 2021), these responses were detected reproducibly across different mice.

## Discussion

We have developed an intermittent awake mice training protocol for concurrent fMRI-pupillometry. Without potential confounding issues of pretreatment of anesthetics or sedatives, the large-scale brain dynamics can be identified with fMRI with real-time pupillometry. We have validated the awake fMRI platform with visual stimulation to show brain activation in the visual areas (VC, SC, and LGd) following light exposure.

Unlike the previous awake rodent fMRI studies (Reed et al., 2013), we compared the pupil dynamics of awake mice through an 8-week training period. It should be noted that numerous awake rodent fMRI studies have applied anesthetics (e.g., isoflurane) before scanning, and the low dosage inhalation of the isoflurane can be quickly cleared after cessation of administration (Harris et al., 2015). This short period of immobility due to anesthesia makes it easier for animal positioning in MRI holders, as well as shimming adjustment before fMRI scanning (Supplementary Table 1). However, the residual effects of anesthetics (Paasonen et al., 2018) remain a potential confounding issue since the administrated volumes and time varied across laboratories. We did not apply any anesthetics, but elongated the training process from 1 week to 8 weeks, showing a well-training mouse tolerating the scanning noise based on the pupil diameter measurement (Figure 3).

The pupil behavior is a reliable marker for characterizing brain state and reflecting the ANS (autonomic nervous system) activities (Bradley et al., 2008; Reimer et al., 2014; Schneider et al., 2016). Previous studies have reported that pupil dilation is modulated through the sympathetic system as a consequence of various cognitive operations, e.g., memory maintenance, mental effort, physiological stress, etc. (Alnaes et al., 2014; Pedrotti et al., 2014; Unsworth and Robison, 2017). In contrast to the highly dilated pupil recorded in the early training phase (1 week), the pupil diameter is significantly reduced in the later phases (5 and 8 weeks). It should be noted that the large variability detected at the early stage across animals could be caused by different stress levels, but the variation of pupil diameter at week 8 of training is also significantly reduced. This reduced variability only indicated that the diameter size across animals is more comparable in well-trained animals, but did not present the resting state pupil variability changes. To further specify the pupil dynamics of a given trial, our future study will need to perform a correlation analysis of fMRI and pupil dynamics acquired during rest. Also, pupil size can be used as an indicator of the sleep state in mice (Yuzgec et al., 2018). It remains challenging to map mouse brain function during sleep with fMRI. The real-time pupillometry with fMRI provides a useful platform to monitor the vigilant states of mice during scanning.

To validate the awake mouse fMRI-pupillometry platform, we delivered visual stimulation to map the brain function in the BOLD-fMRI maps. Consistent with the published reports, BOLD activation maps highlighted several visual pathway-related areas (Niranjan et al., 2016; Dinh et al., 2021). However, it should be noted that the evoked BOLD responses were much less salient than the responses detected in anesthetized animals with alpha-chloralose (Peeters et al., 2001). The peak BOLD signal changes in awake mice were more consistent with the awake human BOLD studies. The altered BOLD peak signal between awake and anesthetized animals could be due to the suppressed spontaneous neuronal activity in the anesthetized brain, which leads to more robust hemodynamic responses coupled with evoked neuronal activity. Our visual stimulation observation is in agreement with several visual pathway-related areas characterized in anesthetized and awake mice (Niranjan et al., 2016; Dinh et al., 2021). A relationship between pupil size and cortical activity, presumably linked to arousal, has been reliably observed in mice during wakefulness and during non-REM (rapid eye movement) sleep (Reimer et al., 2014; McGinley et al., 2015; Yuzgec et al., 2018). These animals also transited between periods of low-population activity (i.e., more desynchronized states) linked to pupil dilations and periods of high-amplitude and low-frequency neuronal firing coupled to constricted pupils. Our simultaneous fMRI and pupillometry recoding not only minimized the effects of anesthesia on neural activity and neurovascular coupling but also provided a reliable marker for characterizing brain state and reflecting the ANS activities based on pupil dynamics. The visual stimuli can potentially alter the brain state of the non-anesthetized mice. We will perform the pupil dynamics with simultaneous fMRI and optical fiber calcium recording, as previously established for anesthetized rodent imaging (Pais-Roldan et al., 2020), to specify the brain state-related changes in our future work.

The intermittent training protocol was extended to 8 weeks in the present study. We observed fast pupil size changes in the early phase of training when head-fixed mice were exposed to scanner noise, but this phenomenon disappeared in the following intermittent exposures (Supplementary Videos 9, 10). The serum corticosterone, an indicator of stress levels, provides a direct measure of stress (King et al., 2005; Tsurugizawa et al., 2020), which in turn can precisely guide experimenters to adjust the step-by-step training procedure in future studies. However, repetitive blood sampling from the same mice remains a concern due to the small circulating blood volume. Therefore, alternative tests (e.g., salivary, urine, or feces testing/counting) might be alternative methods for monitoring the physiological stress.

For the visual stimulation of awake mice, it remains challenging to keep the eyes of mice fixed on the visual targets. In contrast to the eye-tracking system of human fMRI, our real-time pupillometry provided an alternative measure, in particular, when detecting the constriction of pupil diameter. However, the flickering visual stimuli will need to be further modified to optimize the BOLD responses. In addition, since the close distance (<8 mm) between the eye and camera, we will need to replace the camera lens with a longer focal distance to allow a better visual stimulation paradigm for future behavioral studies.

In humans, pathologies such as autism, ADHD, and Alzheimer's disease are associated with atypical pupil size dynamics (Anderson et al., 2006; Reimer et al., 2016; Granholm et al., 2017). Using pupillometry and fMRI could help better understand the link between pupil size and the underlying neuronal activity in mouse models. Although this could be done in humans, mouse fMRI with real-time pupillometry could be more practical for mechanistic studies and therapeutic testing.

## Conclusion

In summary, we report here an awake mouse fMRI training protocol with real-time pupillary without pre-treatment of anesthetics. The visual stimulation-evoked BOLD functional maps in the visual brain areas validate the drug-free awake mouse fMRI platform.

## Data Availability Statement

The original contributions presented in the study are included in the article/Supplementary Material, further inquiries can be directed to the corresponding author.

## Ethics Statement

The animal study was reviewed and approved by Regierungspräsidium, Tübingen, Baden-Württemberg, Germany.

## Author Contributions

XY, YJ, and HZ: research design and data acquisition. HZ and YJ: analysis. HZ: writing–original draft. XY, YJ, and SB-H: writing–review and editing. All authors contributed to the article and approved the submitted version.

## Funding

This research was supported by NIH Brain Initiative funding (RF1NS113278-01, R01 MH111438-01, and U19 NS123717), NIH R01NS120594, R01NS122904, and R21NS121642, NSF grant 2123971, and the S10 Instrument grant (S10 MH124733) to Martinos Center, German Research Foundation (DFG) Yu215/2-1, 3-1, BMBF 01GQ1702, and the internal funding from Max Planck Society.

## Conflict of Interest

The authors declare that the research was conducted in the absence of any commercial or financial relationships that could be construed as a potential conflict of interest.

## Publisher's Note

All claims expressed in this article are solely those of the authors and do not necessarily represent those of their affiliated organizations, or those of the publisher, the editors and the reviewers. Any product that may be evaluated in this article, or claim that may be made by its manufacturer, is not guaranteed or endorsed by the publisher.
