# Supplementary material for: Awake Mouse fMRI and Pupillary Recordings in the Ultra-High Magnetic Field
Source: Front Neurosci. 2022 Jul 6;16:886709. doi: 10.3389/fnins.2022.886709 (PMC9318598; doi:10.3389/fnins.2022.886709)
Supplement: Supplementary file 1 [file Data_Sheet_1.PDF]

Supplementary figures

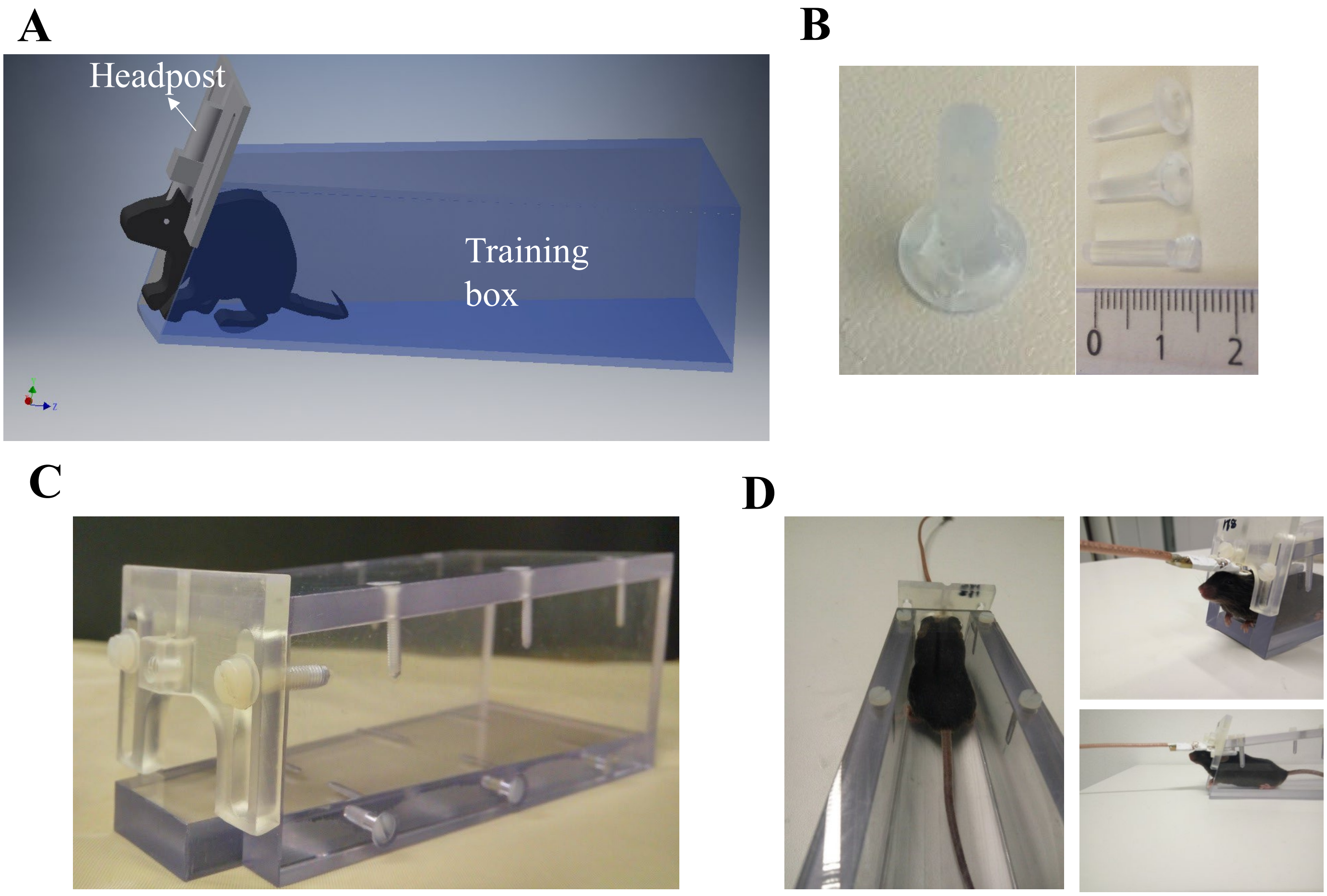

Figure S1. Customized awake restraint system. (**A)** Schematic setups for the awake mouse. (**B**) The 3D printed headpost. (**C**) The 3D printed training box. (**D**) Trained mouse fixation in training box.

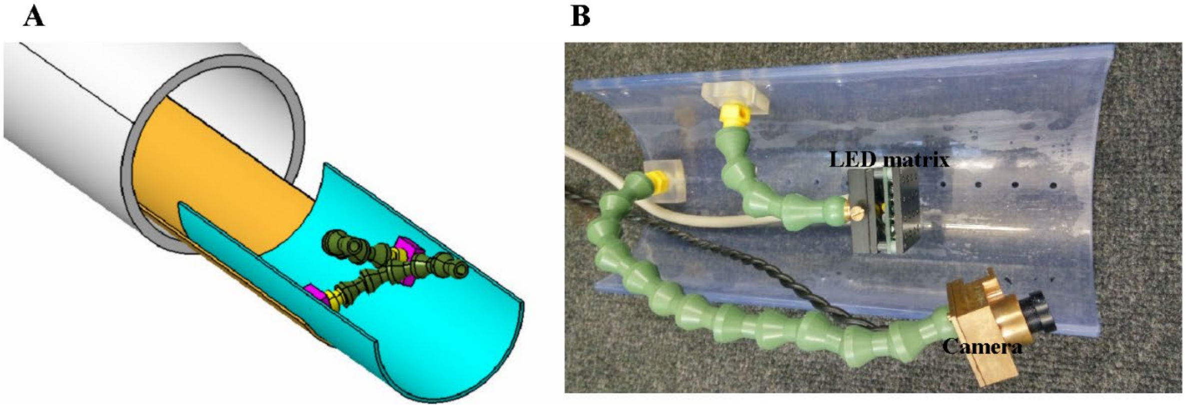

Figure S2. Customized camera holder and LED light matrix for visual stimulation. (**A)** Adjustable camera holder. (**B**) The copper-coded camera and customized LED light matrix.

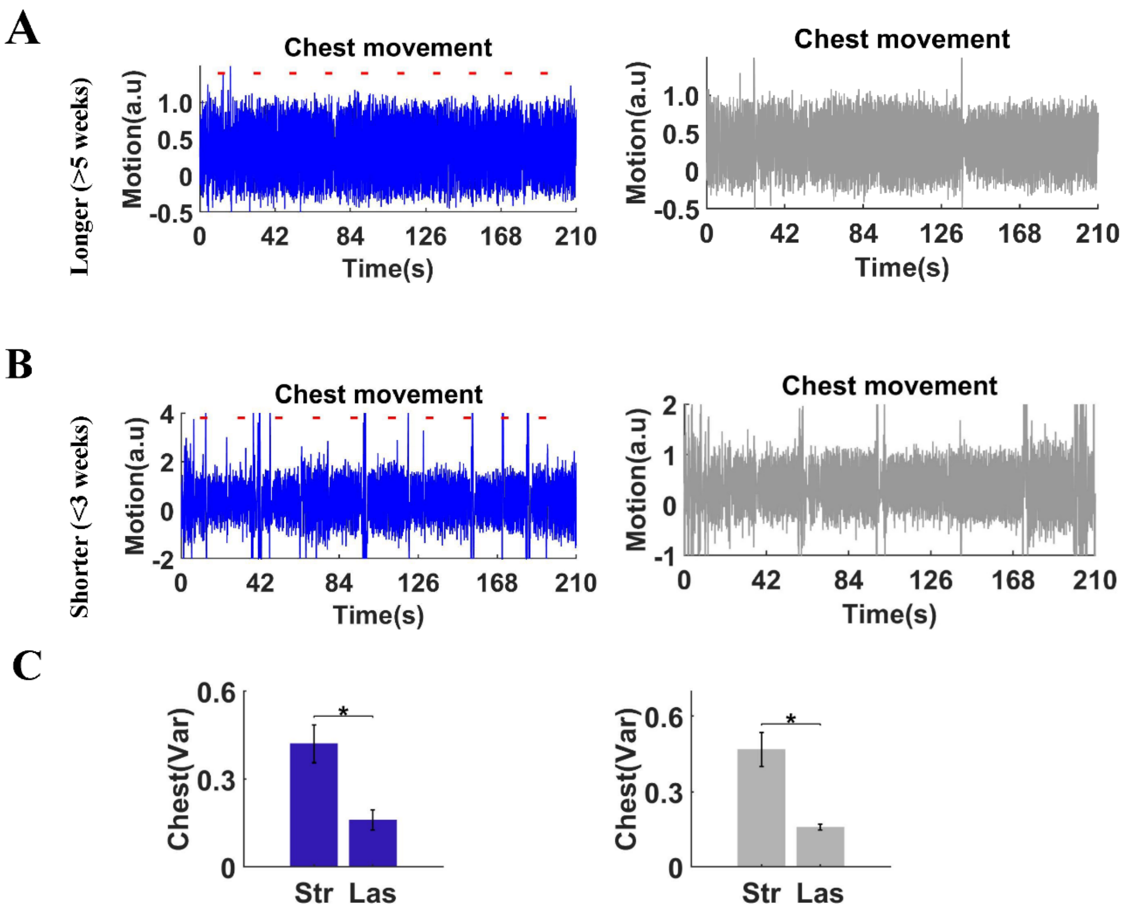

Figure S3. Chest motion assessments for training periods. Representative trials of chest motion assessments from one mouse with (**A**) longer training (>5weeks) and (**B**) shorter training (<3weeks). blue time course with LED light on; gray time course indicates the light off; red dash line is the duration of light on (4s on and16s off). (**C**) The variance of chest movement**.** The variance of chest motion showed a significant decrease after longer acclimation. 4 mice; 52 trials; left, light on; right, light off; Str, very beginning training sessions (~1week); Las, last training sessions(>5weeks); mean± S.D, **p*<0.05.

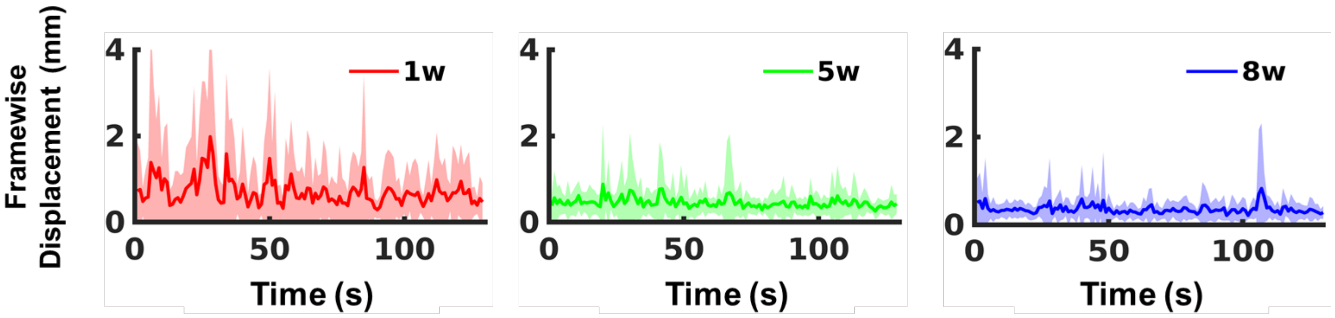

Figure S4. The estimated framewise displacement during intermittent acclimation from different weeks of training (first, fifth, eighth week; shaded regions: mean±S.D., n=6 animals). The framewise displacements (FD) were calculated with 6 motion parameters for each animal based on previously published reports[1; 2].

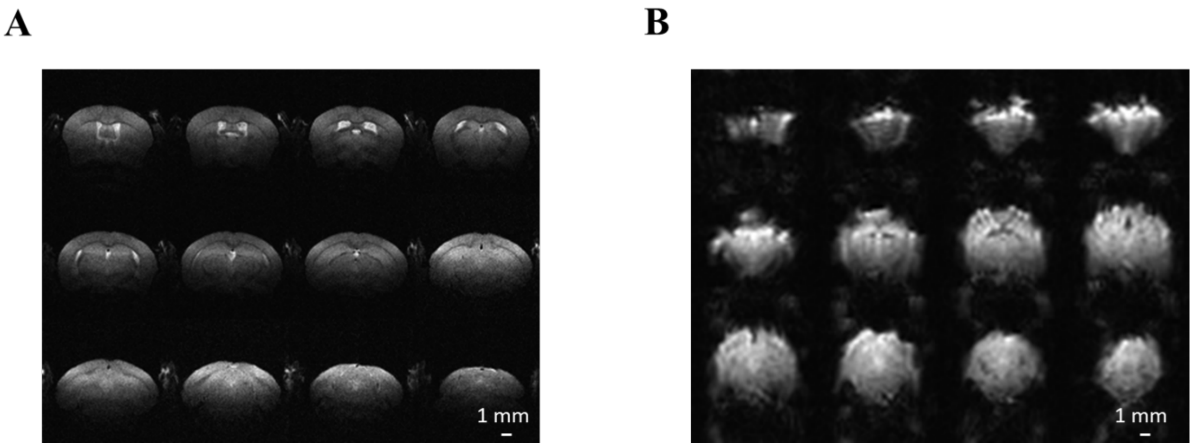

Figure S5. The representative raw anatomical RARE images (**A**) and EPI images (**B**) were acquired from awake mice.

References

[1] J.D. Power, K.A. Barnes, A.Z. Snyder, B.L. Schlaggar, and S.E. Petersen, Spurious but systematic correlations in functional connectivity MRI networks arise from subject motion. Neuroimage 59 (2012) 2142-54.

[2] W.B. Jung, H.J. Shim, and S.G. Kim, Mouse BOLD fMRI at ultrahigh field detects somatosensory networks including thalamic nuclei. Neuroimage 195 (2019) 203-214.
