# Supplementary material for: Awake Mouse fMRI and Pupillary Recordings in the Ultra-High Magnetic Field
Source: Front Neurosci. 2022 Jul 6;16:886709. doi: 10.3389/fnins.2022.886709 (PMC9318598; doi:10.3389/fnins.2022.886709)
Supplement: Supplementary file 2 [file Table_1.DOCX]

**Table S1. Summary of small animal awake fMRI since the year 2000**

| **Ref.** | **Scanner** | **Species** | **Anesthetics** | **Training** | **Setups** | **Ear/Bite bar** | **Stress monitor** | **Motion detection** | **Stimulus** |
| --- | --- | --- | --- | --- | --- | --- | --- | --- | --- |
| (Gutierrez-Barragan et al. 2022) | 7.0T Scanner,  Anat. Image: 0.23×0.23×0.6mm  EPI image: 0.23×0.23×0.6mm | C57BL/6J mice  10 male | None | 4weeks, Day1-3, 10-15min handling, day4, 10min cradle exploration,  Day5, 10min head fixation. Day8-26, mock scanner training, 8min up to 32min | Headpost,  Body and tail were taped to the cradle arc.  Cotton rolls were placed to reduce jaw and forepaw movement. | Unknown | Corticosterone | frame-wise displacements (FD) for motion evaluation | None |
| (Dinh et al., 2021) | 9.4T Scanner,  FLASH Image: 0.059×0.059×0.5mm  EPI image: 0.156×0.1456×0.5mm | C57BL/6 mice,  13 male | 1% isoflurane before training and imaging | 10days, 30min up to 120min, 15min/day increment, for remaining 3days, four mice trained in mock scanner, 3 mice trained in real scanner | Headpost,  Body/neck restrain,  nose cone | Unknown | Unknown | Respiration  Temperature  Chest movement were monitored  frame-wise displacements(FD) for motion evaluation | Visual stim |
| (Tsurugizawa et al., 2020) | 4.7T Scanner,  Anat. Image:  0.125×0.125×1.0mm  EPI Image:  0.2×0.2×1.0mm | 15q dup mice, 25 mice were used for fMRI(including 10 wide type for control ) | 2% isoflurane before training and imaging | 4days, 2 days outside scanner, 2 days inside scanner. 30min at 1^st^ and 60min at 2^nd^. | Headpost  nose mask  ear bar | Ear bar | Corticosterone | Heart rate respiration rate  Six motion parameters (three translation and three rotation) | Ofaction stim |
| (Chen et al., 2020) | 9.4T Scanner,  RARE Image: 0.0625×0.0625×0.4mm  EPI Image: 0.15×0.15×0.4mm  0.1×0.1×0.3mm  0.2×0.2×0.3/0.4mm | C57BL/6 mice  40 male | Unknown | 7days, day1-day4 head fixed with noise,  Day5-day7 with sensory stimulus | Head holder  Kwil-cast  Earphone tube | Ear is fill with the silicone | Unknown | respiratory ate | Whisker auditory  olfactory |
| (Behroozi et al., 2020) | 7.0T Scanner,  RARE Image:  0.23×0.23×0.25mm | Pigeons  Twenty adult Columba livia | Unknown | 3weeks,  10min up to 100 min.  Water deprivation for the whole night before the imaging | plastic pedestal,  head screws,  piezo-electric pressure sensors measuring the dynamic pressure of jaw movements | Unknown | Corticosterone  (ACTH stimulation test) | Respiration rate  heart rate | Color discrimination for Go/no Go task |
| (Desjardins et al., 2019) | 7.0T Scanner,  RARE Image:  0.078×0.039×1.0mm  EPI Image:  0.2×0.2×1.0mm | C57BL/6J, JAX014548,  JAX 005628,  JAX 024109,  16 mice | Anesthetize(<60s) for head fixation | 2 hours/day | Headpost | Ear plugs | Unknown | Camera surveillance | Whisker  optogenetic |
| (Han et al., 2019) | 9.4T Scanner,  RARE Image: 0.0625×0.0625×0.4mm  EPI Image: 0.15×0.15×0.4mm | C57BL/6J mice  8 male | Unknown | water restriction habituation, automatic lick teaching, automatic shaping, GNG task training | Head holder  Kwil-cast | The drum membrane of the middle ear was punctured quickly, and the Kwik-cast was then injected | Unknown | Unknown | olfaction stim for Go/noGO licking |
| (Matsubayashi et al., 2018) | 7.0T Scanner;  RARE Image:  0.075×0.075×0.3mm,  EPI Image:  0.2×0.2×0.5mm | C57BL/6J mice  9 female | Unknown | 5 weeks,2 hours/day, mice underwent laminectomy at the level of the Th9/10 vertebra | Head bar  Body restrain | Unknown | Unknown | Respiration rate | Unknown |
| (Takata et al., 2018) | 7.0 T Scanner,  RARE Image:  0.075×0.075×0.3mm,  EPI Image: 0.2×0.2×0.5mm. | Double transgenic mice  22 male  29 female | Unknown | 7 days,  2 hours/day | optical fiber head-holder | Unknown | Unknown | Unknown | Optogenetics in the left visual cortex |
| (Stenroos et al., 2018) | 7.0 T Scanner,  Anat. Image:  0.097×0.097×0.75mm,  EPI Image: 0.39×0.39×1.5mm. | rat  8 male | 1.5-2% isoflurane before training and imaging | 10 days,  15min up to 45min,  forepaws were attached with masking tape, hind paws were tied together with the tail with tape | Body restrain with cloth | bite bar  shoulder support  pad cheek support  nose cone | Corticosterone | heart rate  respiration | None |
| (Paasonen et al., 2018) | 7.0 T Scanner,  Anat. Image:  0.097×0.097×0.75mm,  EPI Image: 0.39×0.39×1.5mm. | rat  8 male | 1.5-2% isoflurane before imaging | 10 days,  15min up to 45min,  forepaws were secured and hindpaw with tail were secured using masking tape. The body was wrapped with plastic foam sheet | Body restrain with cloth | bite bar  shoulder support  pad cheek support  nose cone  Silicone plugs were inserted into the ear | Corticosterone | Unknown | None |
| (Matsubayashi et al., 2018) | 7.0T Scanner,  RARE Image: 0.075×0.075×0.3mm, EPI Image:  0.2×0.2×0.5mm. | C57BL/6J mice  spinal cord injury  9 female | Unknown | 1 week, 2hurs/day | head-bar | Unknown | Unknown | respiration | None |
| (Ma et al., 2018) | 7.0T Scanner,  Anat. Image: 0.125×0.125×1.0mm, EPI Image:  0.5×0.5×1.0mm. | rat  59 male | 3% isoflurane before imaging | 7 days | bite bar, ears secured by two adjustable ear pads, and nose secured by a nose bar,  head restrainer | Bite bar  Ear bar  Nose bar | Unknown | translational movement | None |
| (Smith et al., 2017) | 4.7 T Scanner,  RARE Image:  0.125×0.125×1.0mm,  EPI Image: 0.5×0.5×1.0mm. | Long Evan rats  42 male | 2% isoflurane before training and imaging | 7days | Headpost,  Body restrain | Unknown | Unknown | Unknown | Nome |
| (Madularu et al., 2017) | 7.0T Scanner,  RARE Image: 0.3×0.3×1.2mm,  Func. Image:  0.3×0.3×1.2mm. | C57BL/6 mice  33 male | 2% isoflurane before training and imaging | 5 days | No headpost, 3D printed restrain system. | Unknown | Respiration rate, Fecal boli | Volume displacement | olfactory stimulus |
| (Chang et al., 2017) | 7.0 Scanner,  Anat. Image:  0.38×0.38×0.5mm,  EPI Image:  0.38×0.38×0.5mm. | rats  22 male | Unknown | 2 weeks, 30mins/day | Head-Post | Unknown | No | respiratory rates | air-puff stimuli to the side surface of their hind paw |
| (Poirier et al., 2017) | 4.7 scanner,  RARE image:  0.125×0.125×1.0mm, EPI image:  0.5×0.5×1.0mm. | adolescent spontaneously hypertensive rat(n=26),  Wistar Kyoto(n=20) and Sprague–Dawley(n=12) | 1% isoflurane before training and imaging | 8 days, 15mins up to 90min, 15 min/day increment | No headpost  head restrainer  body tube | Ear bar  nose clamp | Unknown | Unknown | None |
| (Van Ruijssevelt et al., 2017) | 7.0T Scanner,  EPI Image:  0.25×0.25×0.75mm, RARE Image:  0.07×0.07×0.07mm. | zebra finches  10 male | Unknown | 25days,  10min up to120min | Head fixed,  Body jacket, beak mask and fixation tape. | Unknown | Unknown | Respiration rate | Auditory Stimulus |
| (Berns et al., 2017) | 3.0T Scanner,  Anat. Image:  3.0×3.0×2.5mm,  Func. Image:  3.0×3.0×2.5mm. | Dog  43 both male and female | Unknown | 2 months,  10-15min/day, three days/week, | Unknown | Unknown | Unknown | Unknown | hand signals indicating |
| (Behroozi et al., 2017) | 7.0T Scanner,  EPI Image:  0.15×0.15×0.3mm, RARE Image:  0.47×0.47×1.0mm. | Pigeon  8 Valencian Figurita | Unknown | 10days,  15min up to 60min | plastic pedestal | Unknown | Unknown | Respiration rate | no |
| (Yee et al., 2016) | 7.0T Scanner,  RARE Image:  0.097×0.097×0.7mm  EPI Image:  0.26×0.26×0.7mm | prairie voles  14 male | Unknown | no training procedures,  only restrain in the scanner | Body restrain and nose cone, bite bar, helmet | Bite bar | Unknown | Heart rate | CO2 (5%)  odor |
| (Moore et al., 2016) | 7.0T Scanner,  RARE Image:  0.097×0.097×0.75mm,  EPI Image:  0.26×0.26×0.75mm. | Male Oprm1+/+ and Oprm1−/− mice | 2-3% isoflurane before training and imaging | 4 consecutive days, 30min/day | head in a cushion, ear bars, pressure points used to immobilize the head | Ear bar | Unknown | Unknown | 5% carbon dioxide |
| (Madularu et al., 2016) | 7.0T Scanner,  RARE Image:  0.11×0.11×0.75mm  EPI Image:  0.3125×0.3125×0.75mm | OVX Sprague Dawley  36 rats | 2-3% Isoflurane  before training and imaging | 5 consecutive days, 30mins/day | head in a cushion,  pressure points used to immobilize the head, ear bars, | Ear bar | Unknown | Unknown | None |
| (Kenkel et al., 2016) | 7.0T Scanner,  RARE Image:  0.11×0.11×1.0mm,  EPI Image:  0.3125×0.3125×1.0mm | Adult WT (12) and Fox (10) male Sprague Dawley rats | 2-3% Isoflurane  before training and imaging | 4-5 consecutive days, 30mins/day | Head holder and body tube, no headpost, ear bars, body, paws were tighten in the scanner. | Ear bar | Unknown | Unknown | Odor stimulus |
| (Chang et al., 2016) | 7.0T Scanner,  EPI Image:  0.38×0.38×0.5mm | Sprague-Dawley rats,  10 male | Unknown | 8-10 days, (30min/day) | Headpost,  body restrain | Unknown | corticosterone | Respiration rate | Air-puff to the forepaw |
| (Yoshida et al., 2016) | 7.0T Scanner,  EPI Image:  0.2×0.2×0.5mm | C57BL/6 mice,  8 male | Unknown | 8 days,  2 hours/day | head bar  EMG electrodes | Unknown | ECG  EMG | Heart rate | None |
| (Aksenov et al., 2016) | 9.4T Scanner,  EPI Image:  0.375×0.375×1.0mm | Dutch-belted rabbit  6 female | Unknown | 3-5day, restrained by means of a cloth sleeve | headbolts, electrodes and cannulae were implanted | Unknown | LFP  MUA | Unknown | Optogenetic  Whisker stimulus |
| (Madularu et al., 2015) | 7.0T Scanner,  RARE Image:  0.11×0.11×1.0mm  EPI Image:  0.3125×0.3125×1.0mm | OVX Sprague Dawley rats  27 female | 2-3% Isoflurane  before training and imaging | 5 consecutive days, 30mins/day | head in a cushion  ear bars, pressure points used to immobilize the head | Ear bar | Unknown | Unknown | drug model |
| (Liang et al., 2015b) | 7 T Scanner,  RARE Image:  0.125×0.125×1.0mm  EPI Image:  0.5×0.5×1.0mm | 11 rats | 2% Isoflurane  before training and imaging | 7 days ,  15min up to 90min | four plastic MR compatible screws were fixed into the skull, optic fiber was embedded | Ear bar | Unknown | Unknown | Optogenetics |
| (Liang et al., 2015a) | 4.7 T Scanner,  RARE Image:  0.125×0.125×1.0mm  EPI Image:  0.5×0.5×1.0mm | Long Evan (LE) rats  42 male | 2% Isoflurane  before training and imaging | 7 days,  15min up to 90min | Plexiglas stereotaxic head holder, plastic ear bars, forepaw and hindpaw were loosely taped | Ear bar | Unknown | Unknown | None |
| (Harris et al., 2015) | 7.0 T Scanner,  RARE Image:  0.1×0.1×0.8mm  EPI Image:  0.3×0.3×0.8mm | C57bl/6 mice  44 male | 1–2% Isoflurane  before training and imaging | 5 days, 22min/day,  12-day protocol  6 min up to 22 min | No headpost, limbs were tapped, body were restrained in the body restrainer, nose cone, foam headband | Foam headband into the ear | respiration rate, heart rate,  body movements e.g. leg kicks, arched back)  body weight, Corticosterone | Respiration,  Heart rate,  Body weight,  Body movements,  Corticosterone, | cued fear conditioning Foot shock(0.5mA) |
| (Dilks et al., 2015) | 3.0T Scanner,  Anat. Image:  1.5×1.5×2.0mm,  EPI Image:  3.0×3.0×3.0mm. | 8 Dogs | None | 2-3 months, presentation of images on a computer screen | Unknown | Unknown | Unknown | Unknown | Face processing |
| (Liang et al., 2014) | 4.7 T Scanner,  RARE Image:  0.125×0.125×1.0mm  EPI Image:  0.5×0.5×1.0mm | Long-Evans (LE) adult rats  32 male | 2% Isoflurane  before training and imaging | 7 days,  15min up to 90min | Plexiglas stereotaxic head holder  plastic ear bars, forepaw and hindpaw were loosely taped | Ear bar  Bit bar | anxiety level of rats was assessed by using the EPM test | EPM test | None |
| (Ferris et al., 2014) | 7.0 T Scanner,  RARE Image:  0.097×0.097×0.75mm,  EPI Image:  0.26×0.26×0.75mm. | Wild-type mice (C57B/L6J) (n =5 male, 6 female) and knock in zQ175 HET (n =4 male, 6 female) and HOM (n =5 male, 5 female) mice | 2-3% Isoflurane  before training and imaging | 4 consecutive days, 30min/day | Body tube,  nose cone,  hollow tube | Bite bar | Unknown | displacement | Odor stimulus |
| (Ma et al., 2013) | 3.0 T Scanner,  RARE Image:  0.5×1.0×0.5mm,  EPI Image:  2.0×2.0×2.0mm. | 4 cats | Unknown | Unknown | head-post,  animal chair, | Unknown | Unknown | displacement | Visual stimulus |
| (Johnson et al., 2013) | 7.0 T Scanner,  Anat. Image:  0.11×0.11×1.0mm,  Func. Image:  0.46×0.46×1.0mm. | Sprague–Dawley rats  9 male | Unknown | 10 days cocaine + odor versus no cocaine + no odor training | Chamber,  Electrode,  intravenous catheters, | Unknown | Unknown | Unknown | cocaine-associated odor cue |
| (De Groof et al., 2013) | 7.0 T Scanner,  Anat. Image:  0.085×0.085×0.17mm,  Func. Image:  0.34×0.34×1.0mm. | Pigeons  4 adult | None | three distinct procedures: a)10-30-60min for 3days, b)head fixed 10-20-40min,c) with sounds for 1 week | Cloth jacket, head pedestal; plastic tube; | Unknown | Unknown | displacement | Visual stimuli |
| (Berns et al., 2013) | 3.0 T Scanner,  Anat. Image:  1.5×1.5×2.0mm,  Func. Image:  3.0×3.0×3.0mm. | 15 dogs | None | 2-3months | chin rest, neck coil | Unknown | Unknown | Unknown | Hand signals |
| (Tsurugizawa et al., 2012) | 4.7 T Scanner,  Anat. Image:  0.54×0.54×1.3mm,  Func. Image:  0.27×0.27×1.3mm. | Wistar rats  12 male | 2% isoflurane | 3 days, 30-90min | Body restraint,  head positioner  elastic bands | earplugs | Heart rate respiration rate | Unknown | light stimulation |
| (Desai et al., 2011) | 9.4 T Scanner,  Anat. Image:  0.78×0.78×0.5mm,  Func. Image:  0.1×0.1×0.5mm,  0.2×0.2×0.5mm. | 11 wild-type,  11 transgenic ChR2 mice | 0.7% isoflurane for awake imaging | 3 days, 20-40mins | Headpost, body restraint tube | Unknown | respiration rate | respiration  displacement | optogenetics |
| (Liang et al., 2011) | 4.7 T Scanner,  Anat. Image:  0.125×0.125×1.0mm,  Func. Image:  0.5×0.5×1.0mm. | Long–Evans rat  16 adult male | isoflurane | 8days, 15-90mins | head holder using plastic ear-bars | Ear bar  Bite bar | Unknown | Unknown | None |
| (Zhang et al., 2010) | 4.7T Scanner,  RARE Image:  0.125×0.125×1.0mm,  EPI Image:  0.5×0.5×1.0mm. | Long-Evans (LE) rat  8 adult male | 2% Isoflurane  before training and imaging | 8day,  15min up to 90min | Plexiglas stereotaxic head holder  plastic ear bars, forepaw and hindpaw were loosely taped | Ear bar | Unknown | Unknown | None |
| (Chen et al., 2009) | 4.7T Scanner,  RARE Image:  0.117×0.117×1.0mm,  EPI Image:  0.468×0.468×1.0mm. | 8 female rats | 2% Isoflurane | 3day | Restrainer, headpiece, ear bar, head holder, bite bar, body tube | Ear bar  Bite bar | None | None | water (no odor), lemon scent, and TMT odor |
| (Febo et al., 2008) | 4.7T Scanner,  RARE Image:  0.117×0.117×1.2mm,  EPI Image:  0.468×0.468×1.2mm. | Virgin Sprague-Dawley rats | 2-3% isoflurane | 4day,  90min/session | Headpiece, head holder, Bite bar, ear bar, | Ear bar  Bite bar | None | None | Pup Suckling |
| (Ferris et al., 2008) | 4.7T Scanner,  RARE Image:  0.117×0.117×1.2mm,  EPI Image:  0.468×0.468×1.2mm. | Adult male and female Long-Evans rats | 2–3% isoflurane | 4day,  60min/session | Headpiece, head holder, Bite bar, ear bar, | Ear bar | None | displacement | Drug (SRX251 or fluoxetine) |
| (Duong, 2007) | 4.7T Scanner,  RARE Image:  0.156×0.156×1.5mm,  EPI Image:  0.3125×0.3125×1.5mm. | 7 Sprague–Daley rats | 2% isoflurane | Unknown | restrained with ear-, nose-, tooth-, and shoulder-bars, and a body restraining tub | restrainer with ear-, nose-, tooth-, and shoulder-bars, and a body restraining tub | Respiration rate  Heart rate  blood pressure | Respiration rate  Heart rate  blood pressure | Hypoxia |
| (Chin et al., 2006) | 4.7T Scanner,  Anat. Image:  0.156×0.156×1.25mm,  Func. Image:  0.156×0.156×1.25mm. | Adult male Sprague-Dawley rats | ~3% isoflurane | 4day,  7min up to 60min | Training holder, body tube and headpiece | Unknown | None | None | pharmacological (apomorphine and ABT-594) |
| (King et al., 2005) | 4.7T Scanner,  RARE Image:  0.1×0.1×1.5mm,  EPI Image:  0.2×0.2×1.5mm. | Sprague–Dawley rats  8 male | ketamine and medetomidine being secured | 8 consecutive days, 90min/session | headpiece with blunted ear,  bite bar,  body tube | headpiece with blunted ear,  bite bar,  body tube | respiratory rate and heart rate  corticosterone | corticosterone | None |
| (Febo et al., 2004) | 4.7T Scanner,  Anat. Image:  0.117×0.117×1.2mm,  EPI Image:  0.468×0.468×1.2mm. | Male Sprague-Dawley rats | 2% isoflurane | 3-4 days,  90mins/session | cylindrical head holder, body holder,headpiece, bite bar, ear bar | headpiece, bite bar, ear bar | None | None | cocaine |
| (Sachdev et al., 2003) | 4.7T Scanner,  Anat. Image:  0.23×0.23×1.2mm,  EPI Image:  0.47×0.47×1.2mm. | Long Evans rats  3 male and 2female | Diazepam injection | Body trainer every day for 1 week. after headpost surgery, re-trained for 1 week | felt sleeve, wrapped in a cotton cloth, inserted to the neck into a Plexiglas tube;  Velcro straps | three thread-bearing nylon posts;  lightweight masking tape;  plastic pipe | Unknown | displacement | Whisker stimulus |
| (Kenneth Sicard, 2003) | 4.7T Scanner,  Anat. Image:  0.097×0.097×1.5mm,  EPI Image:  0.039×0.039×1.5mm. | Sprague Dawley rats  15 | 2% isoflurane | None | Secured in a rat restrainer with ear-, nose-, tooth-, and shoulder-bars, a body-restraint tube | Ear bar | blood pressure, heart rate, respiration rate |  | Hypercapnic (CO2) challenges |
| (Peeters et al., 2001) | 7.0T Scanner,  Anat. Image:  0.078×0.156×1.0mm,  EPI Image:  0.156×0.312×1.0mm. | 6 Wistar rats | 5% fluothane,  Atropine injection,  Mivacurium or α-chloralose | None | plexi stereotactic head holder, incisor bar and earplugs | earplugs | None | EEG recording | Electrical forepaw stimulation |
| (Wyrwicz et al., 2000) | 4.7T Scanner,  Anat. Image:  0.156×0.156×2.0mm,  EPI Image:  0.935×0.935×2.0mm. | rabbits (New Zealand white (NZW) or Dutch Belted (DB))  5 female | Unknown | 30min/sessions  1 or 2 sessions | Tied at the neck and tail in soft cloth bag and fastened to an acrylic cradle with velcro straps with four restraining headbolts. | None | None | None | Visual stimulation |

**References:**

Aksenov, D.P., Li, L., Miller, M.J., Wyrwicz, A.M., 2016. Blood oxygenation level dependent signal and neuronal adaptation to optogenetic and sensory stimulation in somatosensory cortex in awake animals. Eur J Neurosci 44, 2722-2729.

Behroozi, M., Helluy, X., Strockens, F., Gao, M., Pusch, R., Tabrik, S., Tegenthoff, M., Otto, T., Axmacher, N., Kumsta, R., Moser, D., Genc, E., Gunturkun, O., 2020. Event-related functional MRI of awake behaving pigeons at 7T. Nat Commun 11, 4715.

Behroozi, M., Strockens, F., Helluy, X., Stacho, M., Gunturkun, O., 2017. Functional Connectivity Pattern of the Internal Hippocampal Network in Awake Pigeons: A Resting-State fMRI Study. Brain Behav Evol 90, 62-72.

Berns, G.S., Brooks, A., Spivak, M., 2013. Replicability and heterogeneity of awake unrestrained canine FMRI responses. PLoS One 8, e81698.

Berns, G.S., Brooks, A.M., Spivak, M., Levy, K., 2017. Functional MRI in Awake Dogs Predicts Suitability for Assistance Work. Sci Rep 7, 43704.

Chang, P.C., Centeno, M.V., Procissi, D., Baria, A., Apkarian, A.V., 2017. Brain activity for tactile allodynia: a longitudinal awake rat functional magnetic resonance imaging study tracking emergence of neuropathic pain. Pain 158, 488-497.

Chang, P.C., Procissi, D., Bao, Q., Centeno, M.V., Baria, A., Apkarian, A.V., 2016. Novel method for functional brain imaging in awake minimally restrained rats. J Neurophysiol 116, 61-80.

Chen, W., Shields, J., Huang, W., King, J.A., 2009. Female fear: influence of estrus cycle on behavioral response and neuronal activation. Behav Brain Res 201, 8-13.

Chen, X., Tong, C., Han, Z., Zhang, K., Bo, B., Feng, Y., Liang, Z., 2020. Sensory evoked fMRI paradigms in awake mice. Neuroimage 204, 116242.

Chin, C.L., Fox, G.B., Hradil, V.P., Osinski, M.A., McGaraughty, S.P., Skoubis, P.D., Cox, B.F., Luo, Y., 2006. Pharmacological MRI in awake rats reveals neural activity in area postrema and nucleus tractus solitarius: relevance as a potential biomarker for detecting drug-induced emesis. Neuroimage 33, 1152-1160.

De Groof, G., Jonckers, E., Gunturkun, O., Denolf, P., Van Auderkerke, J., Van der Linden, A., 2013. Functional MRI and functional connectivity of the visual system of awake pigeons. Behav Brain Res 239, 43-50.

Desai, M., Kahn, I., Knoblich, U., Bernstein, J., Atallah, H., Yang, A., Kopell, N., Buckner, R.L., Graybiel, A.M., Moore, C.I., Boyden, E.S., 2011. Mapping brain networks in awake mice using combined optical neural control and fMRI. J Neurophysiol 105, 1393-1405.

Desjardins, M., Kilic, K., Thunemann, M., Mateo, C., Holland, D., Ferri, C.G.L., Cremonesi, J.A., Li, B., Cheng, Q., Weldy, K.L., Saisan, P.A., Kleinfeld, D., Komiyama, T., Liu, T.T., Bussell, R., Wong, E.C., Scadeng, M., Dunn, A.K., Boas, D.A., Sakadzic, S., Mandeville, J.B., Buxton, R.B., Dale, A.M., Devor, A., 2019. Awake Mouse Imaging: From Two-Photon Microscopy to Blood Oxygen Level-Dependent Functional Magnetic Resonance Imaging. Biol Psychiatry Cogn Neurosci Neuroimaging 4, 533-542.

Dilks, D.D., Cook, P., Weiller, S.K., Berns, H.P., Spivak, M., Berns, G.S., 2015. Awake fMRI reveals a specialized region in dog temporal cortex for face processing. PeerJ 3, e1115.

Dinh, T.N.A., Jung, W.B., Shim, H.J., Kim, S.G., 2021. Characteristics of fMRI responses to visual stimulation in anesthetized vs. awake mice. Neuroimage 226, 117542.

Duong, T.Q., 2007. Cerebral blood flow and BOLD fMRI responses to hypoxia in awake and anesthetized rats. Brain Res 1135, 186-194.

Febo, M., Segarra, A.C., Tenney, J.R., Brevard, M.E., Duong, T.Q., Ferris, C.F., 2004. Imaging cocaine-induced changes in the mesocorticolimbic dopaminergic system of conscious rats. Journal of Neuroscience Methods 139, 167-176.

Febo, M., Stolberg, T.L., Numan, M., Bridges, R.S., Kulkarni, P., Ferris, C.F., 2008. Nursing stimulation is more than tactile sensation: It is a multisensory experience. Horm Behav 54, 330-339.

Ferris, C.F., Kulkarni, P., Toddes, S., Yee, J., Kenkel, W., Nedelman, M., 2014. Studies on the Q175 Knock-in Model of Huntington's Disease Using Functional Imaging in Awake Mice: Evidence of Olfactory Dysfunction. Front Neurol 5, 94.

Ferris, C.F., Stolberg, T., Kulkarni, P., Murugavel, M., Blanchard, R., Blanchard, D.C., Febo, M., Brevard, M., Simon, N.G., 2008. Imaging the neural circuitry and chemical control of aggressive motivation. BMC Neurosci 9, 111.

Gutierrez-Barragan, D., N. A. Singh, F. G. Alvino, L. Coletta, F. Rocchi, E. De Guzman, A. Galbusera, M. Uboldi, S. Panzeri, and A. Gozzi. 2022. 'Unique spatiotemporal fMRI dynamics in the awake mouse brain', Curr Biol, 32: 631-44 e6.

Han, Z., Chen, W., Chen, X., Zhang, K., Tong, C., Zhang, X., Li, C.T., Liang, Z., 2019. Awake and behaving mouse fMRI during Go/No-Go task. Neuroimage 188, 733-742.

Harris, A.P., Lennen, R.J., Marshall, I., Jansen, M.A., Pernet, C.R., Brydges, N.M., Duguid, I.C., Holmes, M.C., 2015. Imaging learned fear circuitry in awake mice using fMRI. Eur J Neurosci 42, 2125-2134.

Johnson, T.R., Smerkers, B., Moulder, J.K., Stellar, J.R., Febo, M., 2013. Neural processing of a cocaine-associated odor cue revealed by functional MRI in awake rats. Neurosci Lett 534, 160-165.

Kenkel, W.M., Yee, J.R., Moore, K., Madularu, D., Kulkarni, P., Gamber, K., Nedelman, M., Ferris, C.F., 2016. Functional magnetic resonance imaging in awake transgenic fragile X rats: evidence of dysregulation in reward processing in the mesolimbic/habenular neural circuit. Transl Psychiatry 6, e763.

Kenneth Sicard, Q.S., Mathew E. Brevard, Ross Sullivan, Craig F. Ferris, Jean A. King, and Timothy Q. Duong, 2003. Regional Cerebral Blood Flow and BOLD Responses in Conscious and Anesthetized Rats Under Basal and Hypercapnic Conditions: Implications for Functional MRI Studies.

King, J.A., Garelick, T.S., Brevard, M.E., Chen, W., Messenger, T.L., Duong, T.Q., Ferris, C.F., 2005. Procedure for minimizing stress for fMRI studies in conscious rats. J Neurosci Methods 148, 154-160.

Liang, Z., King, J., Zhang, N., 2011. Uncovering intrinsic connectional architecture of functional networks in awake rat brain. J Neurosci 31, 3776-3783.

Liang, Z., King, J., Zhang, N., 2014. Neuroplasticity to a single-episode traumatic stress revealed by resting-state fMRI in awake rats. Neuroimage 103, 485-491.

Liang, Z., Liu, X., Zhang, N., 2015a. Dynamic resting state functional connectivity in awake and anesthetized rodents. Neuroimage 104, 89-99.

Liang, Z., Watson, G.D., Alloway, K.D., Lee, G., Neuberger, T., Zhang, N., 2015b. Mapping the functional network of medial prefrontal cortex by combining optogenetics and fMRI in awake rats. Neuroimage 117, 114-123.

Ma, M., Qian, C., Li, Y., Zuo, Z., Liu, Z., 2013. Setup and data analysis for functional magnetic resonance imaging of awake cat visual cortex. Neurosci Bull 29, 588-602.

Ma, Z., Ma, Y., Zhang, N., 2018. Development of brain-wide connectivity architecture in awake rats. Neuroimage 176, 380-389.

Madularu, D., Kulkarni, P., Yee, J.R., Kenkel, W.M., Shams, W.M., Ferris, C.F., Brake, W.G., 2016. High estrogen and chronic haloperidol lead to greater amphetamine-induced BOLD activation in awake, amphetamine-sensitized female rats. Horm Behav 82, 56-63.

Madularu, D., Mathieu, A.P., Kumaragamage, C., Reynolds, L.M., Near, J., Flores, C., Rajah, M.N., 2017. A non-invasive restraining system for awake mouse imaging. J Neurosci Methods 287, 53-57.

Madularu, D., Yee, J.R., Kenkel, W.M., Moore, K.A., Kulkarni, P., Shams, W.M., Ferris, C.F., Brake, W.G., 2015. Integration of neural networks activated by amphetamine in females with different estrogen levels: a functional imaging study in awake rats. Psychoneuroendocrinology 56, 200-212.

Matsubayashi, K., Nagoshi, N., Komaki, Y., Kojima, K., Shinozaki, M., Tsuji, O., Iwanami, A., Ishihara, R., Takata, N., Matsumoto, M., Mimura, M., Okano, H., Nakamura, M., 2018. Assessing cortical plasticity after spinal cord injury by using resting-state functional magnetic resonance imaging in awake adult mice. Sci Rep 8, 14406.

Moore, K., Madularu, D., Iriah, S., Yee, J.R., Kulkarni, P., Darcq, E., Kieffer, B.L., Ferris, C.F., 2016. BOLD Imaging in Awake Wild-Type and Mu-Opioid Receptor Knock-Out Mice Reveals On-Target Activation Maps in Response to Oxycodone. Front Neurosci 10, 471.

Paasonen, J., Stenroos, P., Salo, R.A., Kiviniemi, V., Grohn, O., 2018. Functional connectivity under six anesthesia protocols and the awake condition in rat brain. Neuroimage 172, 9-20.

Peeters, R.R., Tindemans, I., De Schutter, E., Van der Linden, A., 2001. Comparing BOLD fMRI signal changes in the awake and anesthetized rat during electrical forepaw stimulation. Magn Reson Imaging 19, 821-826.

Poirier, G.L., Huang, W., Tam, K., DiFranza, J.R., King, J.A., 2017. Awake whole-brain functional connectivity alterations in the adolescent spontaneously hypertensive rat feature visual streams and striatal networks. Brain Struct Funct 222, 1673-1683.

Sachdev, R.N., Champney, G.C., Lee, H., Price, R.R., Pickens, D.R., 3rd, Morgan, V.L., Stefansic, J.D., Melzer, P., Ebner, F.F., 2003. Experimental model for functional magnetic resonance imaging of somatic sensory cortex in the unanesthetized rat. Neuroimage 19, 742-750.

Smith, J.B., Liang, Z., Watson, G.D.R., Alloway, K.D., Zhang, N., 2017. Interhemispheric resting-state functional connectivity of the claustrum in the awake and anesthetized states. Brain Struct Funct 222, 2041-2058.

Stenroos, P., Paasonen, J., Salo, R.A., Jokivarsi, K., Shatillo, A., Tanila, H., Grohn, O., 2018. Awake Rat Brain Functional Magnetic Resonance Imaging Using Standard Radio Frequency Coils and a 3D Printed Restraint Kit. Front Neurosci 12, 548.

Takata, N., Sugiura, Y., Yoshida, K., Koizumi, M., Hiroshi, N., Honda, K., Yano, R., Komaki, Y., Matsui, K., Suematsu, M., Mimura, M., Okano, H., Tanaka, K.F., 2018. Optogenetic astrocyte activation evokes BOLD fMRI response with oxygen consumption without neuronal activity modulation. Glia 66, 2013-2023.

Tsurugizawa, T., Tamada, K., Ono, N., Karakawa, S., Kodama, Y., Debacker, C., Hata, J., Okano, H., Kitamura, A., Zalesky, A., Takumi, T., 2020. Awake functional MRI detects neural circuit dysfunction in a mouse model of autism. Sci Adv 6, eaav4520.

Tsurugizawa, T., Uematsu, A., Uneyama, H., Torii, K., 2012. Functional brain mapping of conscious rats during reward anticipation. J Neurosci Methods 206, 132-137.

Van Ruijssevelt, L., Hamaide, J., Van Gurp, M.T., Verhoye, M., Van der Linden, A., 2017. Auditory evoked BOLD responses in awake compared to lightly anaesthetized zebra finches. Sci Rep 7, 13563.

Wyrwicz, A.M., Chen, N., Li, L., Weiss, C., Disterhoft, J.F., 2000. fMRI of visual system activation in the conscious rabbit. Magn Reson Med 44, 474-478.

Yee, J.R., Kenkel, W.M., Kulkarni, P., Moore, K., Perkeybile, A.M., Toddes, S., Amacker, J.A., Carter, C.S., Ferris, C.F., 2016. BOLD fMRI in awake prairie voles: A platform for translational social and affective neuroscience. Neuroimage 138, 221-232.

Yoshida, K., Mimura, Y., Ishihara, R., Nishida, H., Komaki, Y., Minakuchi, T., Tsurugizawa, T., Mimura, M., Okano, H., Tanaka, K.F., Takata, N., 2016. Physiological effects of a habituation procedure for functional MRI in awake mice using a cryogenic radiofrequency probe. J Neurosci Methods 274, 38-48.

Zhang, N., Rane, P., Huang, W., Liang, Z., Kennedy, D., Frazier, J.A., King, J., 2010. Mapping resting-state brain networks in conscious animals. J Neurosci Methods 189, 186-196.
